# Probing the Flexibility of Large Conformational Changes in Protein Structures through Local Perturbations

**DOI:** 10.1371/journal.pcbi.1000343

**Published:** 2009-04-03

**Authors:** Bosco K. Ho, David A. Agard

**Affiliations:** Howard Hughes Medical Institute and the Department of Biochemistry, University of California San Francisco, San Francisco, California, United States of America; Stanford University, United States of America

## Abstract

Protein conformational changes and dynamic behavior are fundamental for such processes as catalysis, regulation, and substrate recognition. Although protein dynamics have been successfully explored in computer simulation, there is an intermediate-scale of motions that has proven difficult to simulate—the motion of individual segments or domains that move independently of the body the protein. Here, we introduce a molecular-dynamics perturbation method, the Rotamerically Induced Perturbation (RIP), which can generate large, coherent motions of structural elements in picoseconds by applying large torsional perturbations to individual sidechains. Despite the large-scale motions, secondary structure elements remain intact without the need for applying backbone positional restraints. Owing to its computational efficiency, RIP can be applied to every residue in a protein, producing a global map of deformability. This map is remarkably sparse, with the dominant sites of deformation generally found on the protein surface. The global map can be used to identify loops and helices that are less tightly bound to the protein and thus are likely sites of dynamic modulation that may have important functional consequences. Additionally, they identify individual residues that have the potential to drive large-scale coherent conformational change. Applying RIP to two well-studied proteins, Dihdydrofolate Reductase and Triosephosphate Isomerase, which possess functionally-relevant mobile loops that fluctuate on the microsecond/millisecond timescale, the RIP deformation map identifies and recapitulates the flexibility of these elements. In contrast, the RIP deformation map of α-lytic protease, a kinetically stable protein, results in a map with no significant deformations. In the N-terminal domain of HSP90, the RIP deformation map clearly identifies the ligand-binding lid as a highly flexible region capable of large conformational changes. In the Estrogen Receptor ligand-binding domain, the RIP deformation map is quite sparse except for one large conformational change involving Helix-12, which is the structural element that allosterically links ligand binding to receptor activation. RIP analysis has the potential to discover sites of functional conformational changes and the linchpin residues critical in determining these conformational states.

## Author Summary

Many proteins undergo large motions to carry out their biological functions. The exact nature of these motions is typically inferred from the crystal structures of the protein trapped in different states, which normally constitutes a difficult series of experiments. As molecular dynamics is generally accepted to accurately model the motion of proteins, the promise is that a long enough simulation will generate all the motions of a given protein structure. Unfortunately, current systems run too slowly to simulate all but the smallest motions. To overcome this computational limit, we have developed a molecular-dynamics perturbation method that induces large changes in a protein structure in very short simulation times. The changes correspond to large motions of specific structural elements on the surface of the protein that corroborate well with the canonical motions of several well-characterized proteins. This bodes well for our method to identify, for any given protein structure, structural elements on the surface that might bind drugs, regulate signals, undergo chemical modifications, or become unstructured.

## Introduction

Protein dynamics play a critical role in a wide variety of biological processes such as catalysis, substrate recognition and binding, allosteric regulation and protein stability [1]. The dynamic behavior associated with these biological functions can involve motions as subtle as a sidechain displacement to large-scale rearrangements of entire domains. The timescales and magnitudes of protein conformational dynamics have been revealed by NMR, small angle x-ray scattering, electron microscopy, and single molecule fluorescence [1]. While potentially quite powerful, molecular dynamics (MD) simulations that seek atomic level explanations and predictions of dynamic behavior are limited by the need to sample high energy, transiently populated states in a computationally practical time period. For example, to date the longest MD simulation spans a microsecond [2], whereas biologically important dynamic behavior often occurs on the millisecond (and longer) timescale. Thus, despite the clear importance of protein dynamics to biological function, there is an equally clear need to improve the computational models that seek to populate infrequent and transient states.

Given the time limitations of MD simulations, the feasibility of generating meaningful dynamic information depends critically on the size of the fluctuations. Although small motions such as the gating of the sodium channel [3] can be sufficiently sampled over hundreds of nanoseconds, biologically relevant large-scale conformational changes such as those involved in allosteric regulation [4], molecular motors moving along their filamentous tracks and polymerases moving along DNA [5], require timescales of milliseconds or longer. These timescales are usually inferred from kinetic data or more increasingly from direct observations and single molecule FRET [1]. Where the final state of a large-scale motion can be deduced from crystal structures, MD simulations biased by driving potentials along a pre-defined trajectory can be used to identify critical events along the trajectory [6]. However, such biased simulations cannot be used to predict conformational changes from a single crystal structure. In the absence of alternate conformations, various approximation schemes based on contact analysis, such as guassian network models [7] and FIRST [8], have been devised to generate large domain-level motions of a given protein structure [9],[10].

There is an important class of protein dynamics that lie between the regime of small fluctuations and large domain motions - motions confined to a single structural element moving independently of the rest of the protein, which cannot be readily modeled with contact-based models. Well-studied examples of these intermediate-scale motions show that they are functionally important: the ligand-binding loop on Triosephosphate Isomerase (TIM) fluctuates at a rate of 3×10^4^ s^−1^ [11] where the closed state stabilizes the ligand for catalysis [12]; fluctuations (35 s^−1^) of the Met20 loop of Dihydrofolate Reductase (DHFR) [13] are postulated to be the limiting step of catalysis [14]; and structural rearrangements of Helix-12 of the nuclear hormone receptors, which interacts with bound ligand, is a key determinant of the receptor's allosteric activation [15],[16].

The discovery and modeling of such movable segments are important in understanding the functional dynamics of these and other proteins. As experiments suggest that these motions occur in the microsecond/millisecond range, extraordinarily long MD simulations would be needed to allow the protein to explore the relevant rare fluctuations. To circumvent this practical limit in computation, previous simulations predefined interconversion pathways and applied driving potentials, resorted to high temperatures coupled with manually-chosen backbone constraints to maintain structural integrity [17], or used coarse-grained representations [18],[19],[20]. Motions have also been deduced from normal-mode analysis or quasi-harmonic analysis of nanosecond MD trajectories [21]–[25]. Alternatively, hierarchical loop modeling can identify reasonably accurate low energy conformations of short loops [26]–[28]. As both high temperature simulations and hierarchical loop modeling require prior information about the existence of a flexible region, they are not suitable for prediction purposes. Nevertheless, several systems have been designed to model the local flexibility of a given structure [29]–[32]. Although the flexibility generated by these systems reproduce the NMR S^2^ parameters of various small protein domains, we show that this flexibility does not provide clear evidence of intermediate-scale loop motions.

Here, we propose a new and unbiased approach that is capable of inducing intermediate-scale conformational changes by continually applying a local perturbation throughout a short MD simulation. This method, Rotamerically Induced Perturbation (RIP), was inspired by a perturbation method previously developed in our lab, the Anistropic Thermal Diffusion method [33], which was designed to probe intramolecular signaling within a protein by simulating flow patterns of kinetic energy. In the ATD method, the protein is first cooled to a very low temperature, and then an individual residue is coupled to a 300 K heat bath. For physically interacting residues, a pathway of heat transfer is induced through the protein. The important concept taken from the ATD method is the idea of applying a local perturbation at a single residue in order to generate a deformation in the structure.

To probe larger scale conformational changes, one could imagine simply applying a high temperature “bath” to an individual residue in a protein that has been pre-equilibrated to 300 K, but is otherwise uncoupled from any temperature baths. The applied energy would then be distributed amongst the bond, angle and torsional modes of vibrations in the residue. While this does result in larger perturbations, unfortunately most of the energy is taken up by bond vibrations, which quickly conveys the energy through interconnected covalent bonds along the backbone causing the backbone to unfold at the point of perturbation. In the RIP method, instead of applying a general heat bath to a residue, the perturbation is applied only to the sidechain torsional degrees of freedom, resulting in the rotation of the sidechain χ angles, while the bond lengths remain unperturbed. As this motion is orthogonal to the backbone degrees of freedom; for most residues, the RIP method does not produce significant changes in backbone structure. But for certain residues, the RIP method induces large segments of the protein to move, often by several Ångstroms, in a time period of only 10 picoseconds. As this is a relatively cheap calculation, a global map of deformability can be generated by independently perturbing every residue in the protein.

**Figure 1:**
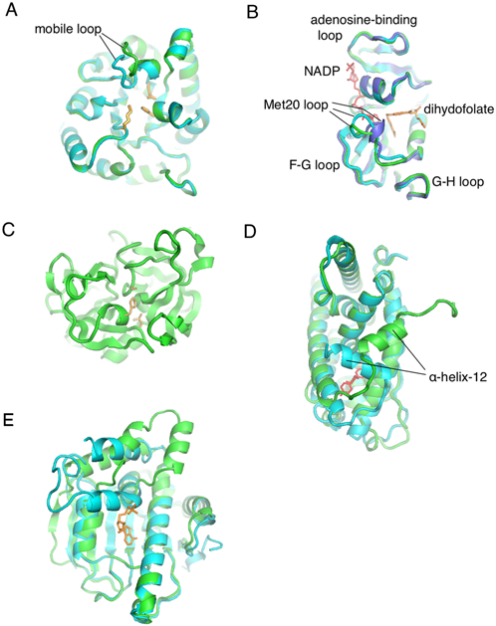
Structures with intermediate-scale motions. (A) Triosephosphate Isomerase (TIM) has a mobile loop that covers the active site (orange). The mobile loop has been crystallized in both an open [8tim] (green) and closed [1TPH] (blue) conformations. (B) Dihydrofolate Reductase (DHFR) pos-sesses the Met20-loop that has been crystallized in three different states - open [1RA2] (green), closed [1RX2] (blue) and occluded [1RX7] (purple). There is experimental evidence that the Met20-loop interacts with the adenosine-binding loop, the F–G loop and the G–H loop. (C) α-Lytic protease (αLP) [1SSX] is the control as it is a kinetically-stable protease (catalytic triad in orange) that does not possess any mobile loops. (D) The Estrogen Receptor (ER) has a highly mobile Helix-12 that covers the ligand (red) binding site. ER has been crystallized in a closed [1QKU] (blue) and open [1QKT] (green) conformation. (E) The N-terminal domain of the chaperone HSP90 (HSP90) has a 23 amino acid lid [2IOR] (green) that undergoes a large conformational change to bind ADP [2IOQ] (blue).

In order to see if the induced perturbations capture information about real proteins, the RIP analysis was applied to five proteins with different dynamics. These include TIM (Figure 1A) and DHFR (Figure 1B), both of which possess loops that have been measured to move on the millisecond/ microsecond timescale; α-lytic protease (αLP) (Figure 1C), a kinetically stable protein that is known to be extremely rigid, which presumably has no mobile loops; the Estrogen Receptor ligand-binding domain (ER) (Figure 1D) in which Helix-12 is known to undergo a large conformation change in response to ligand binding; and finally the N-terminal domain of the chaperone HSP90, which has a large lid that interacts with the active site (Figure 1E). The simulations of these proteins using the RIP method generate deformation maps that characterize the mobility (and lack of mobility) of different segments in the protein structure. Analysis of these deformation maps demonstrate that the simulated dynamic properties of the proteins compare favorably to experiment.

## Results

### Rotamerically Induced Perturbation (RIP)

Previous efforts to induce local perturbations used generic heat baths to apply the perturbation to an individual residue [33],[34]. While sufficient for inducing small-scale changes, we wished to explore applicability to larger perturbations. In our experience, the application of a high-temperature heat bath propagates most of the energy along the backbone, which invariably unfolds the backbone at the point of perturbation. In contrast, the Rotamerically Induced Perturbation (RIP) circumvents the problem of the backbone unfolding by exclusively perturbing the χ angles, generating motions orthogonal to the backbone degrees of freedom.

In the RIP method, the protein is first stripped of ligands and waters, energy minimized, and pre-equilibrated without constraints to 300 K over 10 ps using Amber with GB/SA implicit solvent [35]. To avoid having to modify the Amber source code, the actual RIP calculations are performed by running sets of constant energy 100 fs simulations, which allow the rotamer perturbations to develop, with no other restraints applied. Between each interval, the instantaneous rotational velocity is calculated for each χ angle of the residue being perturbed. The bond and bond angle vibrations are suppressed, and a pure χ rotational velocity of the desired magnitude is applied to the atomic velocities and then the next 100 fs interval begins. Because of the reduced degrees of freedom, imposing a sidechain rotamer kinetic energy of 300 K results in far greater rotational velocities than those in a standard 300 K MD simulation. The direction of rotation is maintained until it exceeds a limit of ±60° from the initial χ angle, upon which the direction of rotation is reversed. This limit restricts the sidechain to exploring the basin surrounding a single rotamer, and allows long sidechains such as MET to effectively explore the landscape of a single rotamer. The resultant motion is that of the sidechain rotating back-and-forth around the initial χ angle. If there are no collisions with other residues, no energy gets transferred. But if there is a collision, a large displacement may be induced in another part of the protein. As the protein is simulated under constant energy during each interval, the transferred energy continues to propagate through the protein, resulting in a slight increase in the overall temperature.

In RIP, the rotational velocities are calculated directly from the rotational inertia of the sidechain. Thus we expect the rotational velocities of the χ angles in different sidechains to be different. For instance, at 300 K the phenyl ring in Phe should rotate more slowly about its χ2 angle than would the methyl-group in Ile about its χ2 angle. In order to demonstrate that RIP generates plausible χ angle behavior, we simulated the 17 amino acids that possess sidechains having χ-angles using a standard MD protocol. The amino acids were capped with methyl groups, and then simulated in AMBER with GBSA for 10 ps using a standard thermal bath at 300 K. The average values of the χ angle rotational velocities are then extracted from these trajectories, providing a reference set of rotational velocities for the χ-angles of each amino acid.

**Figure 2:**
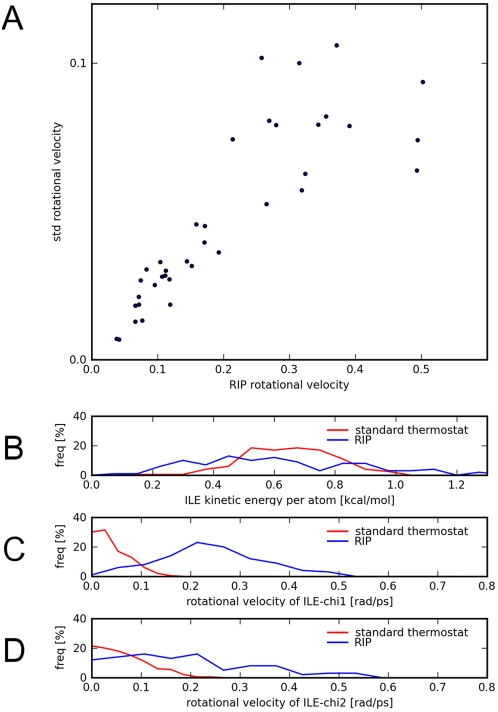
Comparison of the RIP method to standard molecular dynamics using methyl-capped amino acids. (A) The correlation of the average rotational velocities of the χ angles of the 17 amino acids that possess χ angles. Units are in [rad ps^−1^]. In the graph, the Y-axis standard velocities (extracted from standard molecular-dynamics at 300 K) are plotted against the X-axis RIP velocities (in the RIP protocol the kinetic energy at 300 K are effectively transferred to the χ-angle degrees of freedom). The correlation coefficient is 0.84. Detailed comparison for ILE (which has two χ-angles) of the standard molecular-dynamics simulation to the RIP simulation. (B) The distributions of the average kinetic energy per atom are fairly similar. The differences can be seen in the (C) distribution of the χ1 rotational velocities and (D) distributions of the χ2 rotational velocities.

We then performed the RIP method on the same 17 amino acids. The average rotational velocities were extracted from the trajectories of the RIP simulations, and compared to the standard set of rotational velocities (Figure 2A). The Pearson correlation coefficient is 0.84, which shows that the RIP protocol generates the relative differences of the rotational velocities found in the standard simulations. The fit is better for low rotational velocities, which correspond to the motion of the heavier sidechains. The higher rotational velocities, which deviate more from a straight-line fit, correspond to the small sidechains or the ends of the long sidechains.

The differences between a residue perturbed by RIP and a residue regulated with a standard thermostat at 300 K can be shown in greater detail with the results for Ile (Figure 2B–D). As intended by the design of the RIP protocol, the average kinetic energy of the residue perturbed by RIP is equivalent to that in a standard simulation (Figure 2B). The differences can be found in the frequency distributions of the χ angles (Figure 2C and 2D), where RIP induces much higher rotational velocities than does the standard distribution. In the RIP rotational-velocity distributions, χ1 is peaked around 0.21 rad ps^−1^ (Figure 2C) while χ2 is much flatter (Figure 2B). This is due to the smaller weight of the methyl group controlled by χ2, which moves much faster and collides more often with the rest of the Ile amino acid, thus broadening the distribution.

### Mobile Segments in Triosephosphate Isomerase (TIM)

Triosephosphate Isomerase (TIM) has a ligand-binding loop that can close over the active site of the protein. In different crystal structures, this loop is observed in both an open and closed state [36] and NMR relaxation experiments detect loop fluctuations at a rate of 3×10^4^ s^−1^ [11]. TIM typically exists as a dimer in which inter-monomer contacts are mediated through a separate dimer-interface loop.

**Figure 3:**
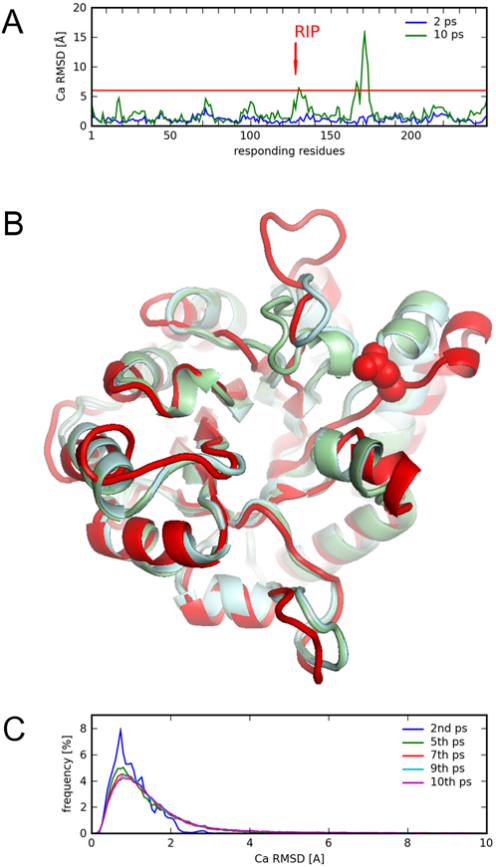
RIP-induced conformation changes of TIM. (A) the Cα RMSD response to the perturbation of RIP on Glu128 (red arrow) shown after 2 ps (blue) and at the end of the 10 ps simulation (green). (B) The 10th ps conformation (red) of the TIM structure due to RIP on Glu128 (red spheres), overlaid over the closed state (blue) and open state (green) of the crystal structures. (C) The frequency distribution of Cα RMSD for all residues from the entire set of perturbations of RIP over every residue in TIM.

The RIP analysis was applied to all residues in the TIM monomer having the open state of the mobile loop (Figure 1A). The simulations were also performed on the closed state of TIM and similar results were generated (discussed below). For each perturbing residue, the response of the protein can be measured by the Cα RMSD of the residues in the protein at the end of the 10 ps simulation. As an example, applying RIP to Glu128, a residue located near the mobile loop of TIM, results in a Cα RMSD deformation response with one large peak at residue 175 (Figure 3A). By overlaying the 10th ps conformation of the RIP simulation over the crystal structures (Figure 3B), it can be seen that the peak of large conformational change at residue 175 corresponds to the ligand-binding loop at residues 167–177. There is also a displacement of the helix near Glu128.

**Figure 4:**
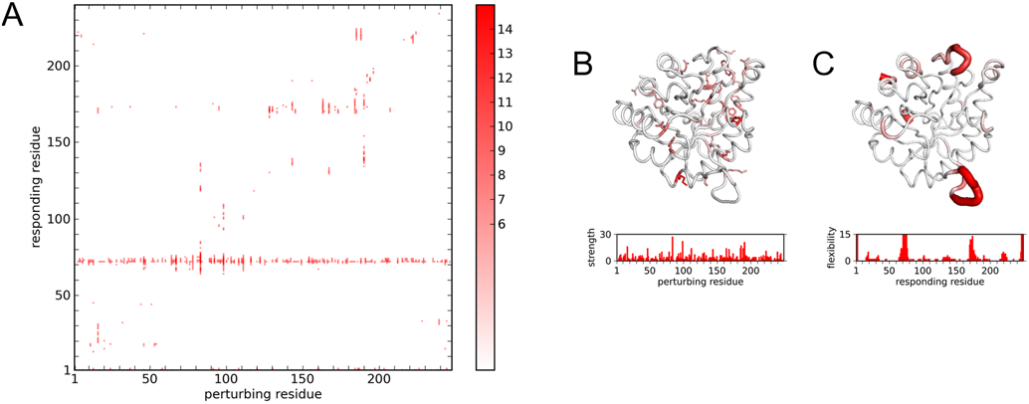
The global Cα RMSD response of TIM to RIP. (A) Each column corresponds to the perturbation of the residue marked by the X-value. Intensity represents Cα RMSD deviation for the 10th ps of simulation above background (>6.0 Å). (B) Perturbation strength histogram and structural linchpins. Structural linchpins are defined if the number of residues with Cα RMSD >6Å induced by a residue is greater than 3σ above the mean. The structural linchpins are mapped onto the structure as sticks. Scale is 0 (white) to 30+ (red). (C) Conversely, local regions that are highly susceptible to perturbation can be identified via a local flexibility histogram and mapped onto the structure. Scale is 0 (white) to 15+ (red).

A global map of deformability in TIM can be constructed by applying RIP to every residue along the entire length of the protein. Each column in the RIP deformation map represents the 10 ps Cα RMSD response to a perturbation of RIP on the residue with sequential numbering on the X-axis (Figure 4A). To facilitate visualization, the actual response is shown on the map only if the responding residue is perturbed by more than a defined threshold. The threshold was chosen by analyzing the probability distribution (Figure 3C) of the Cα RMSD responses in the set of RIP simulations applied to every residue in TIM. This distribution has an average of 1.5Å, a σ of 1.2 Å. The peak of Cα RMSD at 0.8 Å corresponds to the residual motion from the initial pre-equilibration of 300 K. Using a cutoff of 6Å (mean+3.75σ) results in a sparse global map consisting of mostly contiguous segments of deformation (Figure 4A). Lower thresholds result in noisier maps, showing many single-residue deformations.

The first point to note is that there is no systematic response along the diagonal in the RIP deformation map. Residues adjacent to the perturbed residue are not automatically disturbed. This demonstrates the key property of the RIP method: perturbing a sidechain does not systematically disturb the local backbone, unless there is a specific interaction of the perturbed sidechain to the backbone. Consequently, if deformations are observed then they can be directly attributed to the perturbing sidechain.

As is evident from the TIM RIP deformation map, perturbing some residues can induce large changes in the protein while others have virtually no effect. The magnitude of the perturbation inducible by a particular residue can be quantitated by counting the number of residues that respond significantly to the perturbation (above the 6Å threshold). Residues capable of inducing significant perturbations will be referred to as structural linchpins (Figure 4B). While structural linchpins are generally larger amino acids, some smaller amino acids also show up.

Another way of extracting useful information from the RIP deformation map is to quantify the susceptibility of each residue to local perturbation (Figure 4C) by summing the number of above-threshold deformations horizontally across the deformation map (Figure 4A). Unlike the perturbation strength defined above, this local flexibility is a global property based on the entire set of perturbations along the protein chain.

**Figure 5:**
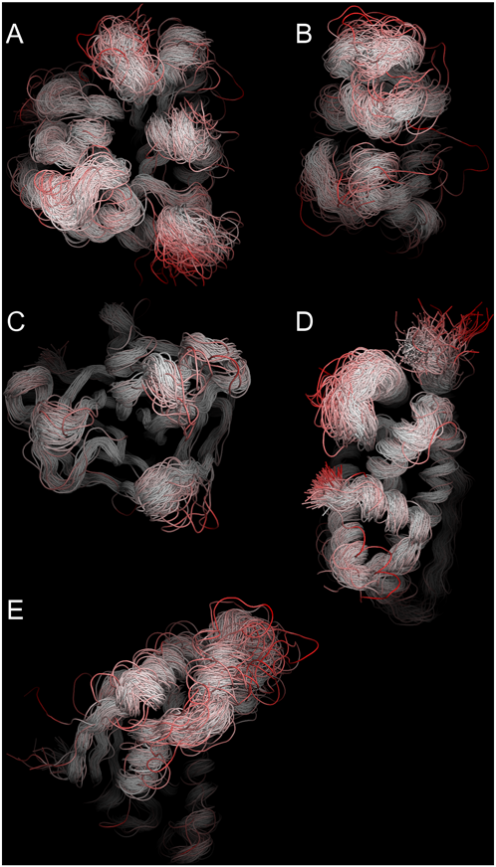
Ensemble of the last conformation of the trajectories of perturbations induced by RIP for (A) TIM, (B) DHFR, (C) αLP, (D) ER, (E) HSP90. Red corresponds to an RMSD deviation of 15 Å or more. These ensembles are used to generate the flexibility histograms.

In TIM, there are two segments with significant local flexibility (Figure 4C). One of the segments of large local flexibility corresponds to a striking horizontal band of deformations at residue 75 in the deformation map (Figure 4A). This segment corresponds to the dimer-interface loop. As deformation of the dimer-interface loop occurs independently of the location of the applied perturbation, a reasonable interpretation is that the dimer-interface loop is an intrinsically mobile loop in the monomer state. In contrast, there is a scattered band of flexibility in the segment corresponding to the ligand-binding loop at residue 175. Flexibility in the ligand-binding loop is induced only by the cluster of structural residues that surround it (Figure 4B). This can be interpreted as the ligand-binding loop being conditionally flexible, i.e., large fluctuations of neighboring residues can readily displace the mobile loop. To illustrate the range of motion induced by the RIP method in the mobile loop of TIM, the entire ensemble of the final conformations of each perturbation is shown (Figure 5A). The range of motion generated by RIP is far greater than the differences between the open and closed conformations of the mobile loop found in the crystal structures (Figure 1A).

### Mobile Elements in Dihydrofolate Reductase

Dihydrofolate Reductase (DHFR) catalyzes the reduction of dihydrofolate by NADP. The protein binds both ligands through the Met20 loop. Crystallography of the enzyme with different ligands has defined three states for the Met20 loop (Figure 1B) suggesting that loop dynamics may be functionally important: i) a closed state where the Met20 loop covers both dihydrofolate and NADP; ii) an occluded state where the Met20 loop packs against the dihdyrofolate but blocks NADP; and iii) an open state where the loop may be disordered in solution (Sawaya and Kraut 1997).

**Figure 6:**
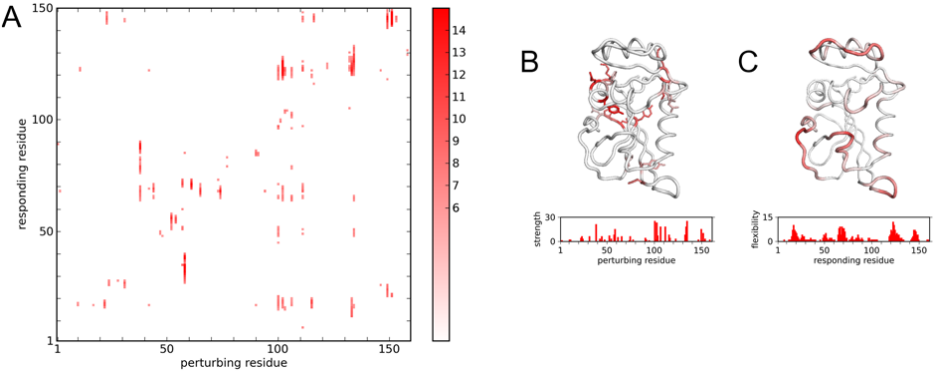
RIP perturbations of Dihydrofolate Reductase (DHFR). (A) RIP deformation map. (B) Structural linchpins and perturbation strength histogram. (C) Local flexibility mapped on structure and in histogram. Colors are as in Figure 4.

Using the RIP analysis, the results of the perturbations can be used to generate a RIP deformation map of DHFR (Figure 6A). From this map, potential structural linchpins can be identified (Figure 6B), and the local flexibility calculated (Figure 6C). The RIP deformation map displays many regions of large conformational changes, dispersed in sequence space. Regions of significant local flexibility (Figure 6C) map to the Met20 loop, the adenosine-binding loop, the F–G loop, and the G–H loop. Experiments suggests that all these loops couple to the activity of the Met20 loop [37],[38].

### The Kinetic Stability of α-Lytic Protease

**Figure 7:**
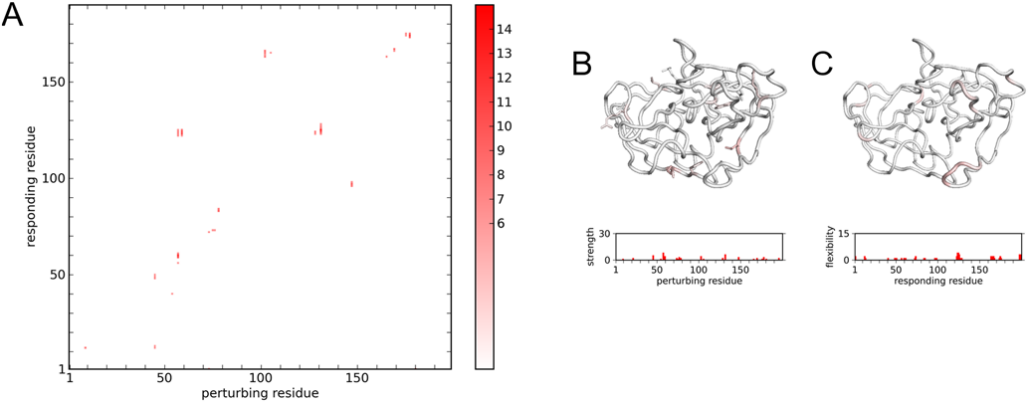
RIP perturbations of α-lytic Protease (αLP). (A) RIP deformation map. (B) Structural linchpins and perturbation strength histogram. (C) Local flexibility mapped on structure and in histogram. Colors are as in Figure 4.

α-Lytic protease (αLP) (Figure 1C) is a kinetically stable protein shown by experiment to be highly resistant to unfolding [39] and to have extraordinarily high hydrogen-exchange protection factors (>10^10^) throughout its large hydrophobic core. The reduced dynamics and absence of mobile segments is believed to be functionally important for minimizing proteolytic destruction [40]. Reflecting these properties, αLP is also found to be extremely stable in standard MD calculations [33], where loops separating secondary structural elements appear to be firmly held by interaction with the body of the protein. This provides a good test for the RIP method, as any large-scale predictions of flexibility would likely be erroneous. In agreement with the known dynamics of αLP, the deformation map for αLP (Figure 7) is remarkably empty; there are only scattered segments of limited deformation, indicating that αLP is particularly insensitive to single residue perturbations and thus, conformationally very stable.

### Motion of Helix-12 of the Estrogen Receptor

The Estrogen Receptor belongs to a family of nuclear receptors that are ligand-inducible transcription factors [15]. The ligand binds in a hydrophobic pocket in the ligand-binding domain of the Estrogen Receptor (ER) that is covered by Helix-12, so that in the bound state the ligand is completely buried. Not only does Helix-12 need to be displaced for ligand entry and exit, but it is also the key allosteric transducer. Crystal structures of ER with bound agonists and antagonists (Figure 1D) reveal that Helix-12 responds to the bound ligand by occupying one of several conformations that serve to either support or block binding of the downstream transcriptional co-activator protein [16]. Clearly, Helix-12 is a functionally-critical mobile segment of the protein although there is no information about the timescale of its motion.

**Figure 8:**
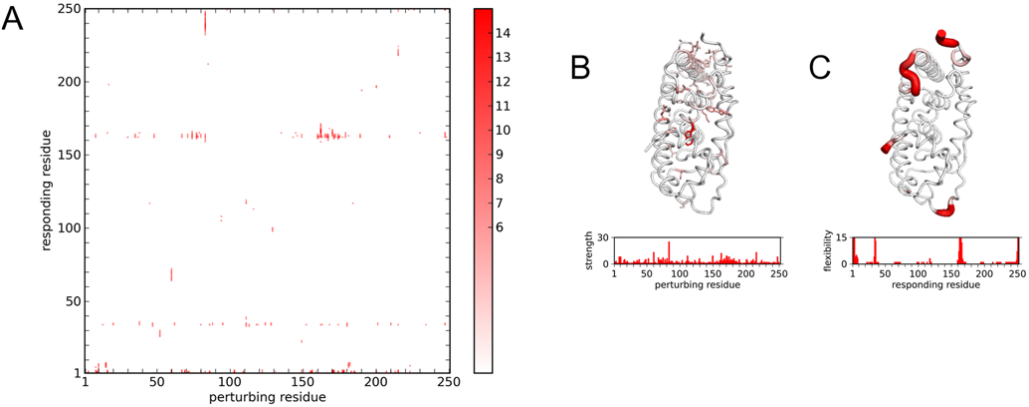
RIP perturbations of Estrogen Receptor (ER). (A) RIP deformation map. (B) Structural linchpins and perturbation strength histogram. (C) Local flexibility mapped on structure and in histogram. Colors are as in Figure 4.

The RIP deformation map of ER (Figure 8A) calculated in the absence of ligand, shows a very limited set of responding distortions with the exception of a horizontal band at residue 162. The horizontal band corresponds to a short loop between the α-helices (Figure 8C). Apart from a large conformational change of a long segment at the C-terminus (residues 232–250), ER appears to be very stable.

**Figure 9:**
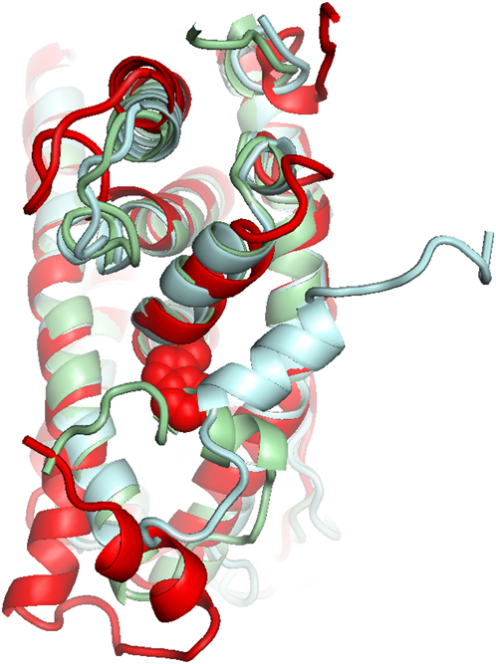
Conformational change in Helix-12 of the Estrogen Receptor ligand-binding domain. (A) Overlay of the crystal structures showing Helix-12 in the closed conformation (blue) and the open conformation (green), which indicates the pivot point between the two structures. In the RIP simulations, perturbation on Trp-83 (red spheres) induces a large conformational change in Helix-12 (red), from the starting conformation of the closed structure (blue), where the hinge of the perturbed motion corresponds to the pivot point of the crystal structures.

The large conformational change in the C-terminus is induced by Trp83, which is the only residue that would qualify as a structural linchpin (Figure 8B). Trp83 has been found to be a highly conserved residue in the nuclear receptor family with the greatest change in the solvent accessible surface area between the apo and holo structures [41], indicating a possible key role in binding Helix-12. The conformation induced by RIP on Trp83 reveals a dramatic cooperative motion in Helix-12, where the α-helix remains intact but moves by 13 Å (red in Figure 9). Although it doesn't move to the same location found in the crystal structure (green in Figure 9), perhaps due to the short simulation, we do see a hinge motion about the same pivot point (blue and green in Figure 9) observed in the crystal structures. Notably, this conformational change does not result in a significant distortion along Helix-12. The ability to generate this kind of large cooperative motion is due in part to the removal of the ligand normally found in the crystal structure for the simulations. In the absence of ligand, Helix-12 covers a cavity and is only weakly bound to the body of the protein. Thus the collisions generated by the perturbation on Trp83 are sufficient to induce the entire helix to move cooperatively.

### Lid Motion of the Chaperone HSP90

**Figure 10:**
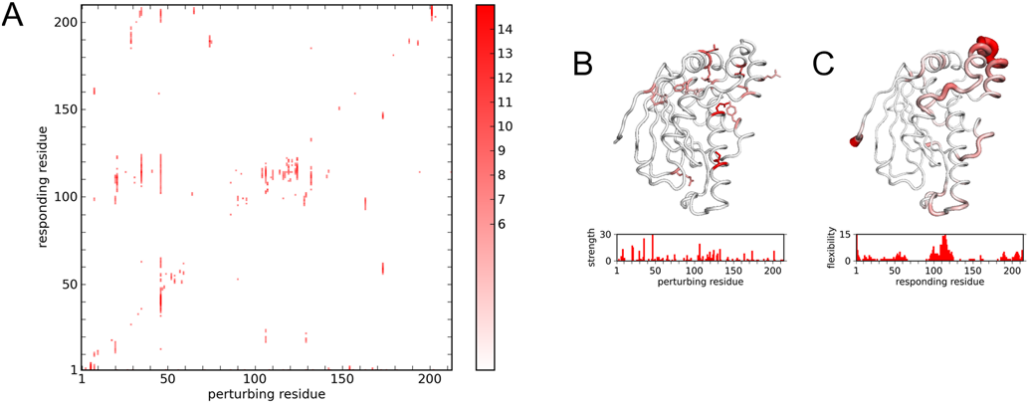
RIP perturbations of HSP90. (A) RIP deformation map. (B) Structural linchpins and per-turbation strength histogram. (C) Local flexibility mapped on structure and in histogram. Colors are as in Figure 4.

Th N-terminal ligand-binding domain of the chaperone HSP90 [42] consists of a active-site lid that is 30 amino acids in length (Figure 1E). The lid is involved in ATP/ADP binding that is associated with the conformational changes of HSP90. The motion of the lid corresponds to an intermediate-scale motion of the lid independent of the body of the protein, as revealed by crystal structures of HSP90 in different ligand-bound states. The RIP deformation map of the HSP90 ligand-binding domain (Figure 10A) in the ADP-bound state, calculated in the absence of ligand, clearly identifies the lid as a highly flexible region. The conformational ensemble of the perturbed structures show large conformational changes in the lid comparable with the scale of motion found in the crystal structures (Figure 5D).

### Comparison of the Flexibility of RIP to Other Systems

**Figure 11:**
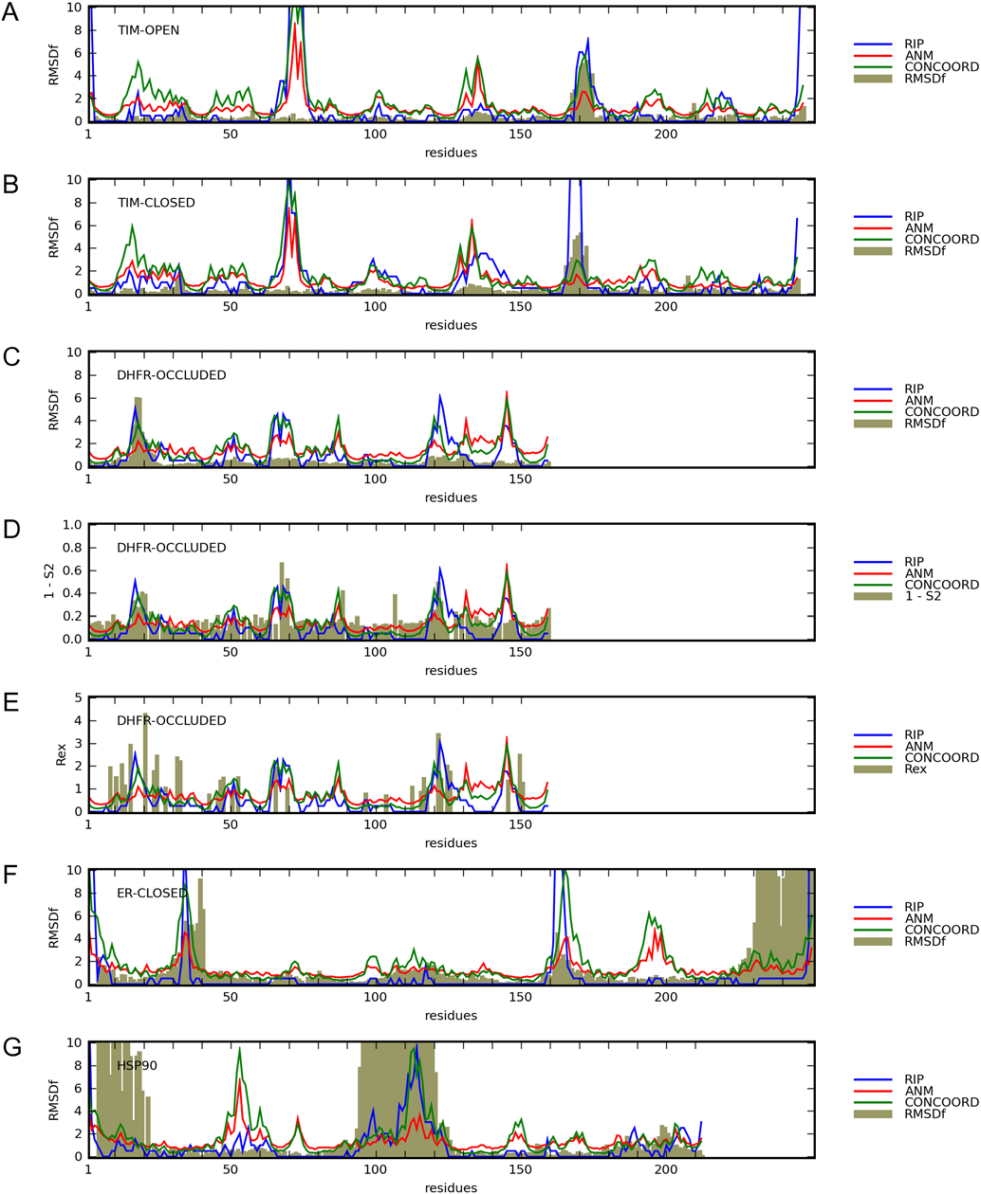
Comparison of the calculated flexibilities to various experimental data. For each graph, an experimental measure (grey) is compared to the flexibility calculated from a structure (labeled on the top left of graph) using RIP (blue), ANM (red) or CONCOORD (green). RMSDf is calculated as the mean of the Cα RMSD of the crystal structures if they are found in different states as shown in Figure 1. (A) Flexibility of TIM in the open conformation compared to the RMSDf. (B) Flexibility of TIM in the closed conformation. Flexibilities from the DHFR occluded structure, compared to (C) RMSDf, (D) S2 parameters and (E) Rex factors. (F) Flexibility of ER in the closed conformation compared to the RMSDf. (G) Flexibility of HSP90 compared to the RMSDf.

There are other systems that calculate protein flexibility from a crystal structure. To compare RIP to these methods (Figure 11), we have calculated the flexibility using the Anisotropic Network Model (ANM) [43] and CONCOORD [32]. We chose ANM as a representative of contact-based network models that generate domain-level motions, where ANM generates a theoretical B-factor for each residue from the sum of the contribution of the lowest modes of oscillations. We chose CONCOORD as a representative of local instability methods, where CONCOORD calculates an RMSDf fluctuation (RMSDf is the averaged Cα RMSD over different conformations of the same structure) at each residue from an ensemble generated from a monte-carlo simulation of the protein using distance constraints derived from a standard force-field. Both methods were chosen largely due to the easy availability of the software implementation. Finally, we compare these flexibilities to various experimental data. For the four proteins with large conformational changes. In the case of TIM, for example, the RMSDf was calculated from the position of the Cα atoms between the open and closed structures (Figure 11A) after they were aligned with the weighted superposition program Theseus [44].

The flexibilities calculated from the open conformation of TIM provide different results with respect to the RMSDf. All three measures of flexibility identify the dimer-interface loop (near residue 65) as flexible even though the RMSDf of the dimer-interface loop is negligible, as both the open and closed conformations exist in the same dimer arrangement in the crystal. For the ligand-binding loop (near residue 165), both RIP and CONCOORD identify elevated flexibility whereas ANM does not. CONCOORD also identifies several other regions as flexible, where there is no corresponding elevated RMSDf values. In contrast, RIP identifies as flexible only the ligand-binding loop and dimer-interface loop. As a further comparison, we show the flexibilities calculated from the closed conformation of TIM (Figure 11B). For the ligand-binding loop, only RIP identifies the loop as intrinsically flexible whereas both ANM and CONCOORD do not.

There exists a rich set of NMR measurements of DHFR that can be used to evaluate the calculated flexibilities of DHFR. From the averaged RMSDf between the open/closed/occluded conformations in crystal structures, only the ligand-binding Met20 displays any large conformation change. We find that both CONCOORD and RIP identify the Met20 loop as flexible whereas ANM does not. This can be contrasted with the S^2^ parameters [22], which measures the backbone ^15^N mobility of DHFR on the picosecond-nanosecond timescale. We find that CONCOORD provides the best agreement to the S^2^ parameters, as would be expected from previous studies [32] (Figure 11D). However, RIP also provides a reasonable match, whereas ANM does not. The similarity between RIP and CONCOORD is surprising as they do not agree in the other proteins. One possibility is that the nanosecond motions of the loops in DHFR correspond to microsecond fluctuations. This can be measured by NMR relaxation exchange factors R_ex_ factors [38] that detect significant backbone fluctuations between distinct states on the microsecond/millisecond timescale. R_ex_ measurements for DHFR identify microsecond dynamics in the Met20 loop, the F–G loop, the adenosine-binding loop and the G–H loop (Figure 11E), suggesting that in the case of DHFR, there is significant overlap between the nanosecond and microsecond dynamics. Coupling between these loops have also been identified in nanosecond MD simulations through cross-correlation and quasi-harmonic analysis [21]–[24].

The large conformational change in Helix-12 of ER, as evident in the large RMSDf values (Figure 11F), provide a difficult challenge for these models of flexibility. There are elevated values of RMSDf in short loops near residue 35 and 165 (Figure 11F), where all three flexibilities finds elevated flexibility. None of the flexibilities identify Helix 12 as a highly flexible region. However, with RIP we can identify one large conformational change by examining the deformability map (Figure 8A), where the conformational change is consistent with the crystal structures (Figure 9). Both ANM and CONCOORD identify elevated flexibility at residue 195, which do not correspond to elevated RMSD values.

The conformational changes in HSP90 is dominated by the motion of the lid, indicated by the large values of RMSDf at residue 110 (Figure 11G). This large conformational change is not identified by ANM but both RIP and CONCOORD identify the lid region as flexible. One advantage with RIP over CONCOORD is that by examining the conformational ensemble of perturbed structures generated by RIP (Figure 5E), we can see that the generated motions are of the order of the RMSDf observed in the crystal structures. Both CONCOORD and ANM identifies a region of elevated flexibility at residue 60 that does not correspond to any elevated region of RMSDf and the large RMSDf in the N-terminal that is involved with inter-domain interactions does not correspond to any calculated flexibility.

In conclusion, we find that the flexibility of RIP identifies only loop motions that correspond to large conformational changes of intermediate-scale motions. In contrast, CONCOORD identifies more regions as flexible, where there is some overlap with the regions identified as flexible by RIP. ANM performs poorly for intermediate-scale motions.

## Discussion

Molecular dynamics (MD) is generally accepted to be an accurate representation of biochemical processes on the molecular level [45]. However, there is a practical upper limit of hundreds of nanoseconds in most MD simulations. Such simulations can only explore small motions in a protein structure whereas many physically interesting processes occur on much longer time-scales. These long times are needed to allow large but rare conformational changes to occur. Perturbation techniques can theoretically be used to quickly generate these rare conformational changes, but there have been many obstacles to applying unbiased perturbation techniques to protein structures using MD. Here, we have developed an unbiased local perturbation method that can generate selective large conformation changes in very short MD simulations.

RIP has several desirable properties that improve upon previous perturbation methods. First, solvation and electrostatics are well treated by the implicit-solvent GBSA method. Second, by driving sidechain rotamers instead of all local atoms, RIP minimizes local backbone distortions, maintaining secondary structures as intact elements. Most perturbations induced by RIP do not result in large-scale distortions of the protein chain, resulting in a sparse map of deformations. Third, RIP can induce large cooperative motions in coherent segments while preserving their local structure, as for example, in Helix-12 in the ER LBD. Fourth, RIP eschews the need for manual restraints or defined trajectories in generating large motions. RIP can thus be applied to any given protein structure. Fifth, RIP is a relatively inexpensive calculation as large Cα RMSD deviations are generated within a short simulation (10 ps), allowing a global analysis to be performed in nanoseconds of simulation time. Since the perturbations induced by each residue are independent, the simulations can be readily performed on a parallel cluster.

As illustrated here, the goal of RIP is to map regions that are readily perturbed and to help discover potential structural linchpins that may dictate local conformation. For example, if a segment is easily deformed by several different perturbations then it is clear that the interactions that bind the segment to the body of the protein are weak. It is found that the local flexibility map, which averages over the global pattern of conformational changes, clearly identifies the loops that have been experimentally determined to be mobile in both DHFR and TIM on the microsecond/millisecond timescale. Furthermore, as revealed by the αLP calculations, the RIP analysis doesn't spuriously find mobile segments where they shouldn't exist. Importantly, RIP can discriminate between proteins that possess intrinsically mobile loops from those that do not.

In comparison, a number of contact-based approximations can deduce large domain-level motions of proteins, such as elastic network models [7] and graph theoretic analysis of the contact network [8]. Essentially contact-based models assume that the network of contacts determine the principal degrees of freedom of the protein. By approximating the contacts as a network of springs, the slow time-scale dynamics of the protein can be deduced from the lowest modes of oscillation of the network of springs. Gaussian network models have modeled the domain-level motions of such large systems as the ribosome [9] and the fluctuations of the capsid of a bacteriophage [10]. It has been shown that the lowest modes of coarse-grain elastic network models reproduce the low frequency modes of more detailed calculations such as normal-mode analysis and quasi-harmonic analysis of MD trajectories over several nanoseconds [46],[47].

Nevertheless, contact-based models cannot detect intermediate-scale motions such as those generated by RIP. In a study of TIM using elastic network models, it was found that the lowest mode of oscillation involved limited motion of the ligand-binding loop [48] where this motion was entangled with other motions in the rest of the protein. In contrast, the liagnd-binding loop in the crystal structures moves independently of the body of the protein (Figure 1A). Independent loop motions are inconsistent with the assumption in contact-based models of the collective motion of a single network of inter-connected springs. When the ligand-binding loop is in the closed state, it forms contacts with the body of the protein. But when the ligand-binding loop opens, the contacts of the loop to the body are broken, resulting in a fundamentally different networks of contacts. Such motions cannot be produced from contact-based models.

Another class of models attempts to identify flexibility through the analysis of local instabilities in a given structure. These models typically generate an ensemble of structures that can be used to calculate instabilities along the protein chain. One approach is COREX that calculates the free-energy of unfolding short segments of a protein structure using an analytical approach [29]. A related approach is the Protein Ensemble Method [30],[31] that explores local unfolding by randomly generating geometric variations of sections of the backbone. Another approach is CONCOORD [32] that generates alternate conformations by monte-carlo exploration of the atoms constrained by distance constraints derived from a standard MD force-field. The flexibility derived from the ensembles generated by PEM and CONCOORD accurately reproduces the NMR S^2^ parameters for several small proteins [30],[32]. The S^2^ parameters of a small protein have been shown to couple strongly to residual dipolar couplings and B-factors that reflect the low-amplitude fluctuations in the picosecond to nanosecond regime [49]. As the flexibility of PEM and CONCOORD correlates well to the S^2^ parameters, this suggests that these instability methods are specifically modeling the low-amplitude fluctuations on the nanosecond regime.

Whilst there is some overlap between CONCOORD and RIP, the flexibility calculated by RIP misses much of the low-amplitude fluctuations that occur on the nanosecond regime as identified by CONCOORD. Instead RIP mainly identifies intermediate-scale motions that occur on the timescale of microseconds or longer. The overlap occurs for loops such as the Met20 loop in DHFR that are mobile on the nanosecond timescale, as revealed by S^2^ parameters, and also on the microsecond timescale, as revealed by the R_ex_ factors. Overlaps between CONCOORD and RIP also occur for intrinsically mobile loop, which are loops that fluctuate >6Å independently of perturbations in short timescales. Apart from intrinsically mobile loops, which can be easily identified from the deformation map as a horizontal band of fluctuations, RIP identifies conditionally flexible regions that correspond to microsecond scale motions, such as the ligand-binding loop in TIM in both the open and closed conformations, and the Helix-12 motion in ER. The flexibilities identified by RIP are more likely to reveal functionally significant conformational changes in a protein structure.

The ability of RIP to generate large conformational changes of several Ångstroms is not due to its ability to sample the rare fluctuations that might occur over a timescale of microseconds or milliseconds. Indeed, because of the non-equilibrium driving conditions, the RIP simulations do not provide any information on the timescale of the simulated motion. Rather it is due to the ability of local perturbations to efficiently explore the strength of contacts that anchor local protein segments. Conformational changes occur only if the perturbation can break the contacts (hydrophobic, polar and hydrogen bonds) that hold these segments to the body of the protein. Although the perturbations are large, as implemented here there is a limit to the extent of perturbation - the overall kinetic energy of the perturbed residue matches that of the same residue equilibrated to 300 K. As such, there is only enough energy to induce conformational changes on segments on the surface of the protein or those near potential packing defects. Importantly this also results in limited distortions within displaced structural elements as in the case of Helix-12 in the ER ligand-binding domain.

It is important to note that the conformational changes generated by the perturbations are artificially large in that they result from large collisions arising from χ angle rotations at velocities far above their normal values. As a consequence, the simulated motions show a large variance in conformations (Figure 5), much more than expected from static snapshots from crystal structures or from NMR ensemble analysis. Although it is unlikely that exactly these perturbed conformations would be generated in a very long equilibrium MD simulation, large RIP-induced conformational changes identify regions where the protein chain can undergo large conformational changes. To obtain more realistic structures, the RIP conformations could be used as starting points for conventional MD simulations or high temperature simulations with manual constraints [50]. The flexible regions identified by RIP may identify potential loops that could be modeled using loop-prediction systems such as PLOP [26],[27] and ROSETTA/BACKRUB [28]. However, as such systems are limited to 12-residue loops, they cannot explore conformations of larger elements such as Helix-12 in ER or the lid in HSP90.

Intriguingly, the motions generated by RIP in DHFR and ER include examples of coupled motions between different mobile segments and ligand-induced structural changes, suggesting that further development of RIP may result in tools to probe mechanisms of allostery. Another possibility is the analysis of the interaction of mobile loops with binding sites, where alterations in surface loop structures can dramatically alter patterns of ligand binding. RIP could provide a computational mechanism for rapid identification of such potentially relevant loops, which might be particularly important for computational ligand screening. Thus RIP followed by MD or loop modeling could provide an efficient means to generate alternate conformations for computational drug discovery.

## Methods

### The Protocol of the RIP Method

The RIP method is implemented as a PYTHON wrapper around the Sander package of AMBER [35] and all analysis code was written in PYTHON. The simulations of the RIP method are run in AMBER, using the PARM96 force-field with an GB/SA implicit-solvent term. To prepare for the simulation, ligands and crystallographic waters are removed from the crystal structure. The structure is then minimized and a Langevin thermometer is applied for a short equilibration at 300 K for 10 ps with a friction constant of 5 ps^−1^.

The standard protocol for a RIP method lasts for 10 ps, which is long enough for large motions to be generated. At the beginning of the RIP method, the equilibrium value of the χ angles of the residue is stored. The run is then broken up into 100 fs intervals where each interval is simulated at constant energy.

Between each interval: (1) the direction of the rotational velocity of each χ angle is stored; (2) the atomic velocities of the residue is set to zero; (3) if the value of the χ angle exceeds 60° of the equilibrium χ value, the direction of the rotational velocity is reversed; (4) the magnitude of each χ rotational velocity is calculated from the sidechain conformation; (5) the χ rotational velocities are transformed into into atomic velocities and added to each atom; (6) the kinetic energy of the residue is scaled to the rotation temperature of 300 K. By scaling the atomic velocities, the kinetic energy of the residue is effectively transfered into the rotational modes of motion. This guarantees that even though the motion is artificially large, the amount of energy in the rotation is not more than would be available for the sidechain at equilibrium, even though this is unlikely to happen.

Between the intervals, a Python module translates the AMBER restart files into a Python object, from which the RIP protocol is used to generate new AMBER restart files for the next interval. Finally, the trajectories of all the intervals are spliced into a single trajectory. Since the modifications are made on the velocities, the coordinate trajectories are continuous.

### Converting Rotational Velocities into Atomic Velocities

In the RIP method, a rotational velocity for each χ angle of a sidechain is calculated at the beginning of every interval. From this rotational velocity, the atomic velocities are generated. To generate the the rotational velocities of the χ angles, each χ angle is assumed to be an independent degree of freedom. Based on the equipartition theorem, each independent χ angle can be assigned an energy E derived from the temperature T. This E is drawn randomly from a Gaussian distribution with mean energy ½kT and standard deviation √(½kT).

To convert a rotational velocity into an atomic velocity, a frame of reference for the axis of rotation must be chosen. As rotational velocities are only defined relative to the axis of rotation; rotations can occur on either end of the axis, and still give the same rotational velocity. Since the purpose of the RIP method is to minimize the motion of the backbone, only the sidechain atoms on the side of the rotation axis away from the backbone are rotated. Consequently, the rotational inertia of each χ angle, I = Σ mr^2^, is calculated as the sum of the moment of inertia of these sidechain atoms, where r is the perpendicular radius of each atom from the χ angle axis of rotation.

To convert E into a rotational velocity ω, the equation of rotational energy E = Iω^2^ is used. This is converted to a tangential velocity v through v = rω. This velocity is applied to the atom along the direction of the tangent to the axis of rotation. The atomic velocities due to each χ angle are then added cumulatively to each atom.

However, the different χ angles of the same sidechain do not represent completely independent degrees of freedom. As such, the final atomic velocities are re-scaled such that the total kinetic energy of the sidechain is E = 3/2 nkT where T = 300 K. This scaling only changes the magnitudes of the rotations and preserves the pure rotation around the χ angles.

### Measuring Rotational Velocities

In the analysis of the RIP simulations, rotational velocities of the χ angles need to be extracted from the trajectories. In the generation of rotational velocities, only atoms that are on the side of the rotation axis of the χ angle away from the backbone contribute to the rotational velocity. Therefore, in the extraction of the rotation velocities, only these atoms are considered. For each atom that fits the criteria, the tangential velocity v to the axis is calculated. This v is converted to a rotational velocity by ω = v/r where r is the perpendicular radius from the axis. As the contributions of each atom to the total rotational velocity of a χ angle depends on its moment of inertia, a weighting (w) for each atom is calculated from the moment of inertia I = mr^2^ of the atom. The weighting is given by w = I / I_total_ where I_total_ is the sum of the I for all the atoms involved in the χ angle. The overall rotational velocity is then given by ω_total_ = Σ wω.
